# A highly pathogenic simian/human immunodeficiency virus effectively produces infectious virions compared with a less pathogenic virus in cell culture

**DOI:** 10.1186/s12976-017-0055-8

**Published:** 2017-04-21

**Authors:** Shoya Iwanami, Yusuke Kakizoe, Satoru Morita, Tomoyuki Miura, Shinji Nakaoka, Shingo Iwami

**Affiliations:** 1 Department of Biology, Kyushu University, Nishi-ku, Fukuoka Japan; 2 Department of Mathematical and Systems Engineering, Shizuoka University, Hamamatsu, Shizuoka Japan; 3 Institute for Frontier Life and Medical Sciences, Kyoto University, Kyoto, Japan; 4 PRESTO, JST, Kawaguchi, Saitama Japan; 5 Institute of Industrial Science, The University of Tokyo, Meguro-ku, Tokyo Japan; 6 CREST, JST, Kawaguchi, Saitama Japan

**Keywords:** Population dynamics model, Parameter estimation, Virus dynamics, Simian/human immunodeficiency virus

## Abstract

**Background:**

The host range of human immunodeficiency virus (HIV) is quite narrow. Therefore, analyzing HIV-1 pathogenesis in vivo has been limited owing to lack of appropriate animal model systems. To overcome this, chimeric simian and human immunodeficiency viruses (SHIVs) that encode HIV-1 Env and are infectious to macaques have been developed and used to investigate the pathogenicity of HIV-1 in vivo. So far, we have many SHIV strains that show different pathogenesis in macaque experiments. However, dynamic aspects of SHIV infection have not been well understood. To fully understand the dynamic properties of SHIVs, we focused on two representative strains—the highly pathogenic SHIV, SHIV-KS661, and the less pathogenic SHIV, SHIV-#64—and measured the time-course of experimental data in cell culture.

**Methods:**

We infected HSC-F with SHIV-KS661 and -#64 and measured the concentration of Nef-negative (target) and Nef-positive (infected) HSC-F cells, the total viral load, and the infectious viral load daily for 9 days. The experiments were repeated at two different multiplicities of infection, and a previously developed mathematical model incorporating the infectious and non-infectious viruses was fitted to the full dataset of each strain simultaneously to characterize the infection dynamics of these two strains.

**Results and conclusions:**

We quantified virological indices including virus burst sizes and basic reproduction number of both SHIV-KS661 and -#64. Comparing the burst size of total and infectious viruses (viral RNA copies and TCID_50_, respectively), we found that there was a statistically significant difference between the infectious virus burst size of SHIV-KS661 and -#64, while there was no significant difference between the total virus burst size. Furthermore, our analyses showed that the fraction of infectious virus among the produced SHIV-KS661 viruses, which is defined as the infectious viral load (TCID_50_/ml) divided by the total viral load (RNA copies/ml), is more than 10-fold higher than that of SHIV-#64 during overall infection (i.e., for 9 days). Taken together, we conclude that the highly pathogenic SHIV produces infectious virions more effectively than the less pathogenic SHIV in cell culture.

**Electronic supplementary material:**

The online version of this article (doi:10.1186/s12976-017-0055-8) contains supplementary material, which is available to authorized users.

## Background

Human immunodeficiency virus (HIV) is able to infect only humans and chimpanzees. This narrow host range makes it difficult for us to establish an appropriate “animal experimental system” for HIV research in order to more fully understand the pathogenesis of HIV infection in vivo. To overcome these difficulties, chimeric simian and human immunodeficiency viruses (SHIVs) that encode HIV-1 Env and are infectious to macaques have been developed [1–3]. For example, a highly pathogenic SHIV strain, SHIV-KS661, which has the *env* gene of HIV-1 89.6 and predominantly uses CXCR4 as the secondary receptor for infection, causes an infection that systemically depletes the CD4^+^ T-cells of rhesus macaques within 4 weeks following infection [4, 5]. On the other hand, a less pathogenic strain, SHIV-#64, which also predominantly uses CXCR4 as the secondary receptor, does not cause severe symptoms in rhesus macaques [6]. In particular, SHIV-#64 infected macaques do not show systemic depletion of CD4^+^ T-cells after infection because viral replication is suppressed by the host immune response [6]. Unlike SHIV-KS661 infection [3, 7], the reduced CD4^+^ T-cell depletion observed in SHIV-#64 infection might lead to better T-cell dependent help for both antibody and CD8^+^ T-cell responses to the virus [6, 8]. So far, we have many SHIV strains that show different pathogenesis in macaque experiments [1–7, 9, 10]. In our previous study [11], we quantified only SHIV-KS661 infection in vitro. However, dynamic aspects of other SHIV strains are not well understood. Quantifying and comparing viral kinetics will provide us with novel insights into the pathogenesis of SHIV strains (and HIV-1) [11–14]. To extend our understanding of the dynamic properties of SHIVs in this study, we focused on two representative strains: SHIV-KS661 and SHIV-#64, and measured the detailed time-course of experimental data in HSC-F cell culture. Using our previously developed method combining in vitro experiments and a mathematical model published in our previous paper [11], we characterized SHIV-KS661 and -#64, and showed a difference between strains based on virological indices including the virus burst sizes and basic reproduction number. Our main finding was that the fraction of infectious virus among the SHIV-KS661 virus progeny is more than 10-fold higher than that of SHIV-#64 during the overall infection in our cell culture. This is a valuable complement to the well-developed in vivo model and can be used to significantly improve our knowledge of SHIV and HIV pathogenesis in vivo.

## Methods

### Cell culture experiment

Our experimental procedures have been previously published [13] but are repeated here for completeness. The virus solution of SHIV-KS661 [5] (or SHIV-#64 [6]) was prepared in a CD4^+^ human T lymphoid cell line, M8166 (a subclone of C8166) [15], and was stored in liquid nitrogen until use. The HSC-F cell line [16] was cultured in a culture medium (RPMI-1640 supplemented with 10% fetal calf serum) at 37 °C and 5% CO_2_ in humidified conditions. Each experiment was performed using two wells of a 24-well plate with a total suspension volume of 2 ml (1 ml per well) and an initial cell concentration of *T*_0_ = 6.46 × 10^6^ cells/ml in each well. Because the initial cell concentration was close to the carrying capacity of a 24-well plate and HSC-F cells replicate slowly, in the absence of SHIV-KS661 (or SHIV-#64) infection the population of target cells changed very little during the timescale of our experiment (data not shown). We therefore neglected the effects of potential regeneration of HSC-F cells in our analysis and in constructing the mathematical model. For virus infection, cultures of HSC-F cells were inoculated 24 h prior to the first infection sampling (*t* = −24 h) at two different multiplicities of infection (MOIs) of 2.0 × 10^−4^ or 2.0 × 10^−5^ 50% tissue culture infectious dose (TCID_50_) per cell of SHIV-KS661 (or SHIV-#64), and were incubated at 37 °C. Four hours after inoculation (*t* = −20 h), the cells were washed to remove the remaining viruses and were replaced into fresh culture medium. The culture supernatant was harvested daily for 10 days (*t* = 0,1,…,9d), and was replaced with fresh medium. On a daily basis, 5.5% of the cells in the culture were harvested to measure the concentration of target and infected cells. Cells were counted by staining with an anti-SIV Nef monoclonal antibody (04-001, Santa Cruz Biotechnology, Santa Cruz, CA) labeled with Zenon Alexa Fluor 488 (Invitrogen, Carlsbad, CA), as previously described [11, 12]. Each harvested supernatant, including 85.4% of the culture virus was stored at -80 °C, and the amount of viral RNA was quantified by RT-PCR, as previously described [11, 12, 17]. The infectious viral load was measured by TCID_50_ assay in HFC-S cell cultures using 96-well flat bottom plates at cell concentrations of 1.0 × 10^6^ cells/ml. The titer of the virus was determined as described by Reed and Muench [18].

### Mathematical model

We employed the following mathematical model considering the infectious and non-infectious viruses, which was developed in our previous paper [11]:

1 $$ {T}^{\prime }(t)=-\beta T(t){V}_I(t)- d T(t), $$

2 $$ {I}^{\prime }(t)=\beta T(t){V}_I(t)-\delta I(t), $$

3 $$ {V_I}^{\prime }(t)= f{p}_{RNA} I(t)-{c}_{50}{V}_I(t)-{c}_{RNA}{V}_I(t), $$

4 $$ {V}_{NI}^{\prime }(t)=\left(1- f\right){p}_{RNA} I(t)+{c}_{50}{V}_I(t)-{c}_{RNA}{V}_{NI}(t), $$

where *T*(*t*) and *I*(*t*) are the concentration of target (susceptible: Nef-negative HSC-F cells) and infected (virus-producing: Nef-positive HSC-F cells) cells per ml of medium, respectively, and *V*_*I*_ (*t*) and *V*_*NI*_ (*t*) are the concentration of RNA copies of infectious and non-infectious virus per ml of medium, respectively. Parameters *d*, *δ*, *c*_*RNA*_ and *β* represent the death rate of target cells, the death rate of infected cells, the degradation rate of viral RNA and the rate constant for infection of target cells by virus, respectively. We assumed that each infected cell releases *p*_*RNA*_ virus particles per day (i.e., *p*_*RNA*_ is the viral production rate of an infected cell), of which a fraction *f* are infectious and 1 - *f* are non-infectious. Infectious virions lose infectivity at rate *c*_50_, becoming non-infectious. A detailed explanation of Eqs. (1,2,3 and 4) can be found in our previous paper [11].

### Mathematical model for data analysis

In the experiments discussed above, the viral load was measured either as the total count of extracellular virions, expressed as RNA copies/ml (two RNA copies equals one virion) and measured via quantitative PCR, or as a relative concentration of extracellular infectious virions, expressed as TCID_50_/ml (proportional to the concentration of infectious virions) and measured via virus titration in cell cultures. To analyze our cell culture experimental datasets, the time-course of the concentration of Nef-negative and Nef-positive HSC-F cells and the viral loads consisting of RNA copies/ml and TCID_50_/ml, for SHIV-KS661 and -#64, we transformed Eqs. (1,2,3 and 4) into the following scaled model [11]:

5 $$ {T}^{\prime }(t)=-{\beta}_{50} T(t){V}_{50}(t)- d T(t)-\mu T(t), $$

6 $$ {I}^{\prime }(t)={\beta}_{50} T(t){V}_{50}(t)-\delta I(t)-\mu I(t), $$

7 $$ {V_{RNA}}^{\prime }(t)={p}_{RNA} I(t)-{c}_{RNA}{V}_{RNA}(t)- c{V}_{RNA}(t), $$

8 $$ {V_{50}}^{\prime }(t)={p}_{50} I(t)-{c}_{50}{V}_{50}(t)-{c}_{RNA}{V}_{50}(t)- c{V}_{50}(t), $$

where *V*_*RNA*_(*t*) = *V*_*I*_(*t*) + *V*_*NI*_(*t*) is the total concentration of viral RNA copies, *V*_50_(*t*) = α*V*_*I*_(*t*) is the infectious viral load expressed in TCID_50_/ml, and *α* is the conversion factor from infectious viral RNA copies to TCID_50_. Parameters *β*_50_ = *β*/*α* and *p*_50_ = *αfp*_*RNA*_ are the converted infection rate constant and production rate of infectious virus, respectively. For each of the daily measurements of the cells and virus concentration, the concentration of Nef-negative and Nef-positive HSC-F cells must be reduced in our model by 5.5% and the viral loads (RNA copies and TCID_50_) by 85.4% to account for the experimental harvesting of cells and virus. These losses were modeled in Eqs. (5,6,7 and 8) by approximating the sampling of cells and virus as a continuous exponential decay, yielding a rate of *μ* = 0.057 per day for cell harvest (i.e., log(1 − 0.055)) and *c* = 1.93 per day for virus harvest (i.e., log(1 − 0.854)). The rates at which SHIV-KS661 and -#64 virions lose infectivity, *c*_50_ = 0.869 and 0.992, per day and the rate at which their viral RNA degrades, *c*_*RNA*_ = 0.091 and 0.160, per day were each estimated directly in separate experiments [13]. These parameter values are summarized in Table 1.

**Table 1:** Parameter values and derived quantities for the in vitro experiment

Parameter name	Symbol	Unit	SHIV-KS661		SHIV-#64	
			Value	95% CI	Value	95% CI
Calculated parameters for the continuous approximation of cell and virus harvest						
Harvest rate of target and infected cells	*μ*	day^-1^	0.057	―	0.057	―
Harvest rate of total and infectious virus	*c*	day^-1^	1.93	―	1.93	―
Fitted parameters from separate experiments						
Rate of virion infectivity loss	*c*_50_	day^-1^	0.869	0.598–1.141	0.992	0.866–1.117
Degradation rate of virion RNA	*c*_*RNA*_	day^−1^	0.091	0.029–0.152	0.160	0.051–0.269
Parameters obtained from simultaneous fit to full in vitro dataset						
Rate constant for infections	*β*_50_	(TCID_50_/ml・day)^−1^	1.49 × 10^5^	7.97 × 10^−6^–2.46 × 10^5^	1.47 × 10^4^	8.86 × 10^−5^–2.36 × 10^4^
Death rate of target cells	*d*	day^−1^	6.95 × 10^−3^	3.89 × 10^4^–5.15 × 10^2^	2.47 × 10^−3^	1.09 × 10^3^–5.75 × 10^3^
Death rate of infected cells	*δ*	day^−1^	1.48	1.20–1.81	1.56	1.23–1.96
Production rate of total virus	*p*_*RNA*_	RNA copies・day^−1^	3.74 × 10^4^	1.94 × 10^4^–6.35 × 10^4^	4.15 × 10^4^	2.67 × 10^4^–6.07 × 10^4^
Production rate of infectious virus	*p*_50_	TCID_50_・day^−1^	0.322	0.164–0.593	0.028	0.0156–0.0426
Quantities derived from fitted values						
Viral burst size (total)	*p*_*RNA*_/*δ*	RNA copies	2.52 × 10^4^	1.39 × 10^4^–4.08 × 10^4^	2.65 × 10^4^	2.03 × 10^4^–3.43 × 10^4^
Viral burst size (infectious)	*p*_50/_ *δ*	TCID_50_	0.215	0.127–0.351	0.018	0.0104–0.0280
Malthusian coefficient	*M*	―	3.08	2.82–3.52	2.59	2.37–2.80
Basic reproductive number (without removal)	*R*_0_	―	19.6	16.5–23.6	13.4	11.4–15.7
Basic reproductive number (with removal)	*R*_0_^*^	―	6.25	5.29–7.51	4.84	4.13–5.62

### Parameter estimation

**Fig. 1:**
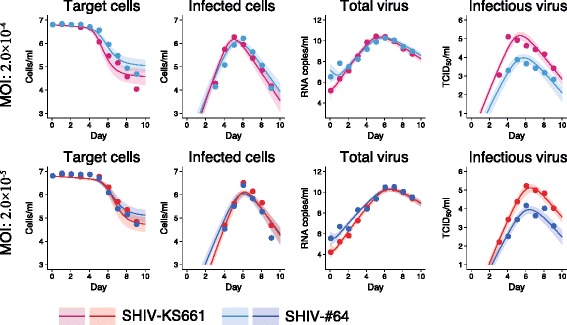
Dynamics of SHIV-KS661 and -#64 infection in HSC-F cells. HSC-F cells were inoculated with SHIV-KS661 or -#64 at two different multiplicities of infection (i.e., 2.0 × 10^−4^ or 2.0 × 10^−5^ TCID_50_ per cell) in cell cultures. Panels show the time-course of experimental data (log scale) for the concentration of Nef-negative and Nef-positive HSC-F cells and the viral loads consisting of RNA copies/ml and TCID_50_/ml for SHIV-KS661 and -#64, respectively. The shadow regions correspond to 95% posterior predictive intervals, the solid curves give the best-fit solution (mean) for Eqs. (5,6,7 and 8) to the time-course dataset. All data for each strain were fitted simultaneously

**Fig. 2:**
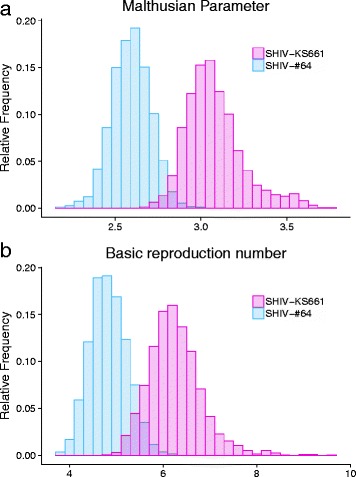
Distribution of Malthusian coefficients and basic reproduction numbers for SHIV-KS661 and -#64 in HSC-F cells. The distributions of the Malthusian coefficients, *M*, and the basic reproduction numbers, *R*_0_^*^, that were calculated from the estimated parameter distributions are shown in (**a**) and (**b**), respectively, for SHIV-KS661 (*red*) and -#64 (*blue*) strains. These indices of *M* and *R*_0_^*^ for SHIV-KS661 are significantly different from those for SHIV-#64, as assessed by the repeated bootstrap *t*-test

**Fig. 3:**
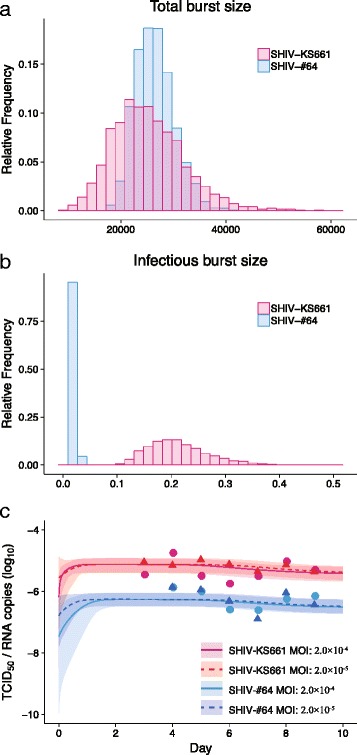
Dynamics of infectious virus for SHIV-KS661 and -#64 in HSC-F cells. The distributions of the total viral burst size (RNA copies), *p*_*RNA*_/*δ*, and the infectious burst size (TCID_50_), *p*_50_/*δ*, that were calculated from the estimated parameter distributions are shown in (**a**) and (**b**), respectively, for SHIV-KS661 (*red*) and -#64 (*blue*) strains. The time-course of the infectious viral load as a proportion of the total viral load are calculated in (**c**). The shadow regions correspond to 95% posterior predictive intervals, the *solid* and *dashed curves* give the best-fit solution (mean) for *V*_50_(*t*)/ *V*_*RNA*_(*t*) to the time-course datasets. The *pink* (*solid curve and round points*) and *red* (*dashed curve and triangular points*) colors correspond to 2.0 × 10^−4^ and 2.0 × 10^−5^ MOI of SHIV-KS661, while the *light blue* (*solid curve and round points*) and blue (*dashed curve and triangular points*) colors correspond to 2.0 × 10^−4^ and 2.0 × 10^−5^ MOI of SHIV-#64, respectively

The Bayesian inference model adopted in this paper assumes measurement error to follow normal distribution with mean zero and unknown variance (error variance). A distribution of error variance is also inferred with the Gamma distribution as its prior distribution. Posterior predictive parameter distributions as an output of Markov chain Monte Carlo (MCMC) computation represents parameter variability. In relating our daily experimental measurements at time *t* = 0,1,…,9 day to our mathematical models, we define *t* = 0 as the time of our first experimental measurements, i.e. when *T*(0), *I*(0), *V*_*RNA*_(0), and *V*_50_(0) are measured. As such, by time *t* = 0, some cells (*T*) have become infected (*I*), and while the inoculum virus has been washed, some new virus (*V*_50_) will have been produced by the newly infected cells. The remaining five free model parameters (*β*_50_, *d*, *δ*, *p*_*RNA*_, *p*_50_) along with eight initial (*t* = 0) values for the variables (four at each of the two MOI values) were determined. Distributions of the model parameters and the initial values were inferred directly by MCMC computations. On the other hand, distributions of the basic reproduction numbers and the other quantities were calculated from the inferred parameter sets (see Figs. 2 and 3a,b for graphical representation). A set of computations for Eqs. (5,6,7 and 8) with estimated parameter sets gives a distribution of outputs (virus load and cell density) as model predictions. To investigate the variation of model predictions, global sensitivity analyses were performed. The range of possible variation is drawn in Figs. 1 and 3c as 95% credible intervals. Technical details of MCMC computations and repeated bootstrap *t*-test are summarized in Additional file 1.

## Results and Discussion

### Data fitting to quantify SHIV-KS661 and -#64 infection in HSC-F cells

Correctly estimated parameter sets with possible variation are required to reproduce model predictions for quantification of SHIV dynamics [19–21]. However, point estimation of the model parameter set by a conventional ordinary least square method does not capture possible variations of kinetic parameters and model predictions (see Additional file 2: Figure S1, Additional file 3: Table S1, and Additional file 4: Table S2). To assess the variability of kinetic parameters and model predictions, we performed Bayesian estimation for the whole dataset using MCMC sampling (see Methods and Additional file 1), and simultaneously fitted Eqs. (5,6,7 and 8) to the concentration of Nef-negative and Nef-positive HSC-F cells and the viral loads consisting of RNA copies/ml and TCID_50_/ml with different MOI values, for SHIV-KS661 and -#64, respectively, as described in [19–21]. We used the parameters in Additional file 3: Table S1 and Additional file 4: Table S2 as the initial estimate of MCMC sampling.

The remaining five free model parameters (*β*_50_, *d*, *δ*, *p*_*RNA*_, *p*_50_) along with eight initial values for the variables were determined by fitting the model to the data. Experimental measurements, which were below the detection limit, were excluded in the fitting. The estimated parameters of the model and derived quantities are given in Table 1, and the estimated initial values are summarized in Table 2. The typical behavior of the model using these best-fit parameter estimates is shown together with the data in Fig. 1 for SHIV-KS661 and -#64, which reveals that Eqs. (5,6,7 and 8) describe these in vitro data very well. The shadowed regions correspond to 95% posterior predictive intervals, the solid lines give the best-fit solution (mean) for Eqs. (5,6,7 and 8), and the dots show the experimental datasets.

**Table 2:** Fitted initial (*t* = 0) values for the in vitro experiment

Variable	Unit	Fitted initial value at MOI of			
		SHIV-KS661		SHIV-#64	
		2 × 10^−4^	2 × 10^−5^	2 × 10^−4^	2 × 10^−5^
*T*_*j*_(0)	cells/ml	6.31 × 10^6a^	6.31 × 10^6a^	6.24 × 10^6 a^	6.24 × 10^6a^
*I*_*j*_(0)	cells/ml	17.9 (5.41–38.8)^b^	0.802 (0.358–1.47)^b^	40.3 (5.78–1.22 × 10^2^) ^b^	8.71 (1.08–29.4)^b^
*V*_*RNAj*_(0)	RNA copies/ml	2.15 × 10^5^ (2.58 × 10^4^–8.30 × 10^5^)^a^	2.45 × 10^4^ (3.45 × 10^3^–9.98 × 10^4^)^a^	1.66 × 10^7^ (1.09 × 10^6^–8.25 × 10^7^)^a^	4.90 × 10^5^ (6.94 × 10^4^–1.82 × 10^6^)^a^
*V*_50*j*_(0)	TCID_50_/ml	0.0729 (6.94 × 10^−4^–0.432)^a^	0.0304 (2.16 × 10^−3^–0.121)^a^	0.124 (5.11 × 10^−4^–0.609)^a^	0.0468 (9.71 × 10^−3^–0.124)^a^

^a^ *T*_*j*_(0) are fixed

^b^best fit value (95% CI)

### Malthusian parameter and basic reproduction number for SHIV-KS661 and -#64 in HSC-F cells

The fitness (or speed) of a SHIV strain in cell culture is described by the Malthusian coefficient, *M*, defined for Eqs. (5,6,7 and 8) [20–22]. Here, the Malthusian coefficient is

$$ M=\frac{-\left(\delta +\mu +{c}_{RNA}+{c}_{50}+ c\right)+\sqrt{{\left({c}_{RNA}+{c}_{50}+ c-\delta -\mu \right)}^2+4{p}_{50}{\beta}_{50} T(0)}}{2}. $$

Using estimated parameter distributions, we calculated the distribution of *M* for SHIV-KS661 and -#64 in Fig. 2a (see Methods). The mean values of *M* for SHIV-KS661 and -#64 are significantly different (Fig. 2a; *p* = 6 × 10^−6^ by repeated bootstrap *t*-test, see Additional file 1) at 3.08 (95% confidence interval (CI): 2.82–3.52) and 2.59 (95% CI: 2.37–2.80), respectively (Table 1). This difference in the value of *M* between SHIV-KS661 and -#64 might explain the earlier and more rapid viral load expansion (i.e., peak viral load) and the systemic depletion of the CD4^+^ T-cells in infected rhesus macaques within a few weeks of SHIV-KS661 infection [3–7], implying a strong relationship between SHIV replication ability and its disease severity.

The other important quantity characterizing virus dynamics is the basic reproduction number, *R*_0_^*^, which is the average number of newly infected cells produced from any one infected cell, under conditions where most of the target cells are uninfected [11, 12, 20–22]. In Eqs. (5,6,7 and 8), the basic reproduction number is defined as

$$ {R}_0^{*}={\beta}_{50}{p}_{50} T(0)/\left\{\left(\delta +\mu \right)\left({c}_{R NA}+{c}_{50}+ c\right)\right\}. $$

Similarly, we calculated the distributions of *R*_0_^*^ for SHIV-KS661 and -#64 (Fig. 2b). The mean value of *R*_0_^*^ for SHIV-KS661 is 6.25 (95% CI: 5.29–7.51), which is significantly higher (Fig. 2b; *p* = 4.2 × 10^− 5^ by the repeated bootstrap *t*-test) than that of SHIV-#64 (4.84; 95% CI: 4.13–5.62) (Table 1). Again, this difference in the value of *R*_0_^*^ between SHIV-KS661 and -#64 implies that the highly pathogenic SHIV strain more efficiently causes systemic CD4^+^ T-cell depletion. In Additional file 5: Figure S2, we calculated the distribution of the basic reproduction number without the effects of removal, *R*_0_ = *β*_50_ *p*_50_ *T*(0)/*δ*(*c*_*RNA*_ + *c*_50_), defined in our previous paper [14] and observed the same trend.

### Viral burst size for SHIV-KS661 and -#64 in HSC-F cells

Interestingly, we found the viral fitness (or speed), *M*, and the infection potential, *R*_0_^*^, of SHIV-KS661 were significantly higher than those of SHIV-#64 in HSC-F cells (Fig. 2). Hereafter, to quantitatively explain a possible mechanism that there are significant differences between SHIV strains, we investigated and compared the total and infectious virus burst sizes (i.e., *p*_*RNA*_/*δ* and *p*_50_/*δ*, respectively) [11]. In Fig. 3a and b, we calculated the distributions of burst sizes for SHIV-KS661 and -#64 using all accepted MCMC estimated parameter values. Surprisingly, we found that there was a statistically significant difference in the infectious burst size, measured in TCID_50_, between SHIV-KS661 and -#64 (Fig. 3b; *p* = 2.5 × 10^− 5^ by the repeated bootstrap *t*-test), while there was no significant difference in the total burst size, measured in viral RNA copies (Fig. 3a; *p* = 0.34 by the repeated bootstrap *t*-test). This implies that the highly pathogenic SHIV produces more infectious virions compared with the less pathogenic strain. In addition, we compared experimental measurement of the infectious viral load as a proportion of the total viral load (i.e., the fraction of the infectious virus among the produced viruses), with the time evolution of the proportion calculated by our mathematical model (i.e., *V*_50_(*t*)/*V*_*RNA*_(*t*)) in Fig. 3c. Despite MOI values and time post infection, we confirmed that the experimental proportions were shown to be steady state values, and the model predictions converged with those values for both SHIV strains. Of great interest was the more than 10-fold difference in the ratios between SHIV-KS661 and -#64 during the overall infection. This strongly supports our above hypothesis that the highly pathogenic SHIV effectively produces infectious virions, which leads to earlier and more rapid viral load expansion and the systemic depletion of the CD4^+^ T-cells in SHIV-KS661-infected rhesus macaques.

## Conclusion

We inoculated HSC-F cells with SHIV-KS661 or -#64 at two different MOIs (i.e., 2.0 × 10^−4^ or 2.0 × 10^−5^ TCID_50_ per cell) and measured in detail the time-course of the experimental data (i.e., the concentration of Nef-negative and Nef-positive HSC-F cells and the viral loads consisting of RNA copies/ml and TCID_50_/ml). Using our previously developed method combining in vitro experiments and a mathematical model in our previous paper [11], we quantified and compared the basic reproduction numbers and the virus burst sizes for the SHIV-KS661 and -#64. Based on our quantitative analysis, we concluded that SHIV-KS661 effectively produces infectious virions compared with SHIV-#64 in the HSC-F cell culture. SHIV-KS661 contains mutations in the *pol* and *env* genes, which are considered to lead SHIV-KS661 to produce more virus and have higher infectivity than SHIV-#64 (data not shown), which is consistent with our conclusion. Although we used the Malthusian parameter, *M*, and the basic reproduction number, *R*_0_^*^ to compare the differences in viral characteristics here, we need to consider evasion of the acquired immune response in infected rhesus macaques to further understand the differing pathogenesis displayed by these viruses, such as systemic CD4^+^ T-cell depletion in SHIV-KS661 infections. Since we cannot observe the effect of the immune response in our cell culture experiments, it will be necessary to analyze the in vivo data from SHIV infected macaques in future work.

## Additional files

Additional file 1: Technical details of MCMC computations. (PDF 49 kb)

Additional file 2: Figure S1. Dynamics of SHIV-KS661 and -#64 infection in HSC-F cells using nonlinear least-squares regression. We simultaneously fit Eqs. (5,6,7 and 8) to the concentrations of Nef-negative and Nef-positive HSC-F cells and the viral loads consisting of the RNA copies/ml and TCID_50_/ml for both MOIs of SHIV-KS661 and -#64, respectively, in A and B using nonlinear least-squares regression that minimizes the sum of squared residuals (SSR). Experimental measurements below the detection limit were excluded when 2 computing the SSR. The solid curves give the best-fit solution for Eqs.(5,6,7 and 8) and the dots are corresponding to the time-course dataset (log scale): blue, red, green, and yellow represent Nef-negative and Nef-positive HSC-F cells and the viral loads consisting of the RNA copies/ml and TCID_50_/ml, respectively. The estimated parameters of the model and derived quantities are given in Additional file 3: Table S1, and the estimated initial values are summarized in Additional file 4: Table S2. (PDF 146 kb)

Additional file 3 Table S1. Parameter values for the in vitro experiment by the nonlinear least squared methods. (PDF 57 kb)

Additional file 4: Table S2. Fitted initial (t = 0) values for the in vitro experiment by the nonlinear least squared methods. (PDF 64 kb)

Additional file 5: Figure S2Distribution of basic reproduction numbers without removal for SHIV-KS661 and -#64 in HSC-F cells. The distributions of the basic reproduction numbers without the effect of removal, $R$_0_=$\beta$_50_ $p$_50_ $T$(0)/$\delta$($c$_RNA_+$c$_50_), that were calculated from the estimated parameter distributions are shown for SHIV-KS661 (red) and -#64 (blue) strains. The basic reproduction number for SHIV-KS661 is significantly different from that for SHIV-#64, as assessed by the repeated bootstrap *t*-test. (PDF 117 kb)

## Abbreviations

HIV: Human immunodeficiency virus
MCMC: Markov chain Monte Carlo
MOI: Multiplicity of infection
SHIV: Simian and human immunodeficiency virus
TCID_50_: 50% tissue culture infectious dose
