# Supplementary material for: A highly pathogenic simian/human immunodeficiency virus effectively produces infectious virions compared with a less pathogenic virus in cell culture
Source: Theor Biol Med Model. 2017 Apr 21;14:9. doi: 10.1186/s12976-017-0055-8 (PMC5401468; doi:10.1186/s12976-017-0055-8)
Supplement: Supplementary file 1 — Technical details of MCMC computations. (PDF 49 kb) [file 12976_2017_55_MOESM1_ESM.pdf]

Supplementary file1: Technical details of MCMC computations

Package FME [1] in Statistical Software R [2] was employed to infer posterior predictive parameter distributions. The delayed rejection and Metropolis method [3] as a default computation scheme of FME was applied to perform MCMC computations. MCMC computations for parameter inference were implemented by using the pre-defined function modMCMC() in package FME, as introduced in Material and Methods. Convergence of Markov chains to a stationary distribution is required to ensure parameter sets are sampled from a posterior distribution. Only the last 15000 among 50000 chains were used as burn-in. The convergence of last 15000 chains was manually checked with figures produced by package coda [4], a collection of diagnostic tools of MCMC computation. The 95% credible interval drawn as a shadow region in each panel of **Fig. 2** is produced from 100 randomly chosen inferred parameter sets and corresponding model predictions. We employed a bootstrap *t*-test [5] to quantitatively characterize the difference of the Malthus coefficient M and basic reproduction number R_0^* between SHIV-KS661 and -#64. Total 10000 parameter sets were sampled with replacement from the posterior predictive distributions to calculate the bootstrap *t*-statistics for M and R_0^*. To avoid potential bias due to sampling, the bootstrap t-test was performed 100 times repeatedly. The average of the computed *p*-values was finally used as an indicator of difference.

SI References

1. K. Soetaert and T. Petzoldt. Inverse Modelling, Sensitivity and Monte Carlo Analysis in R Using Package FME, Journal of Statistical Software, 33(3) 1-28 (2010).
2. R Core Team (2014). R: A language and environment for statistical computing. R Foundation for Statistical Computing, Vienna, Austria.
3. H. Haario, M. Laine, A. Mira, and E. Saksman. DRAM: Efficient Adaptive MCMC, Statistics and Computing, 16, 339-354 (2006).
4. M. Plummer, N. Best, K. Cowles, and K. Vines. CODA: Convergence Diagnosis and Output Analysis for MCMC, R News, vol 6, 7-11 (2006).
5. B. Efron, and R.J. Tibshirani, An introduction to the bootstrap. CRC press (1994).
