# Supplementary material for: A highly pathogenic simian/human immunodeficiency virus effectively produces infectious virions compared with a less pathogenic virus in cell culture
Source: Theor Biol Med Model. 2017 Apr 21;14:9. doi: 10.1186/s12976-017-0055-8 (PMC5401468; doi:10.1186/s12976-017-0055-8)
Supplement: Supplementary file 2 — Dynamics of SHIV-KS661 and -#64 infection in HSC-F cells using nonlinear least-squares regression. We simultaneously fit Eqs. (5,6,7 and 8) to the concentrations of Nef-negative and Nef-positive HSC-F cells and the viral loads consisting of the RNA copies/ml and TCID50/ml for both MOIs of SHIV-KS661 and -#64, respectively, in A and B using nonlinear least-squares regression that minimizes the sum of squared residuals (SSR). Experimental measurements below the detection limit were excluded when 2 computing the SSR. The solid curves give the best-fit solution for Eqs.(5,6,7 and 8) and the dots are corresponding to the time-course dataset (log scale): blue, red, green, and yellow represent Nef-negative and Nef-positive HSC-F cells and the viral loads consisting of the RNA copies/ml and TCID50/ml, respectively. The estimated parameters of the model and derived quantities are given in Additional file 3: Table S1, and the estimated initial values are summarized in Additional file 4: Table S2. (PDF 146 kb) [file 12976_2017_55_MOESM2_ESM.pdf]

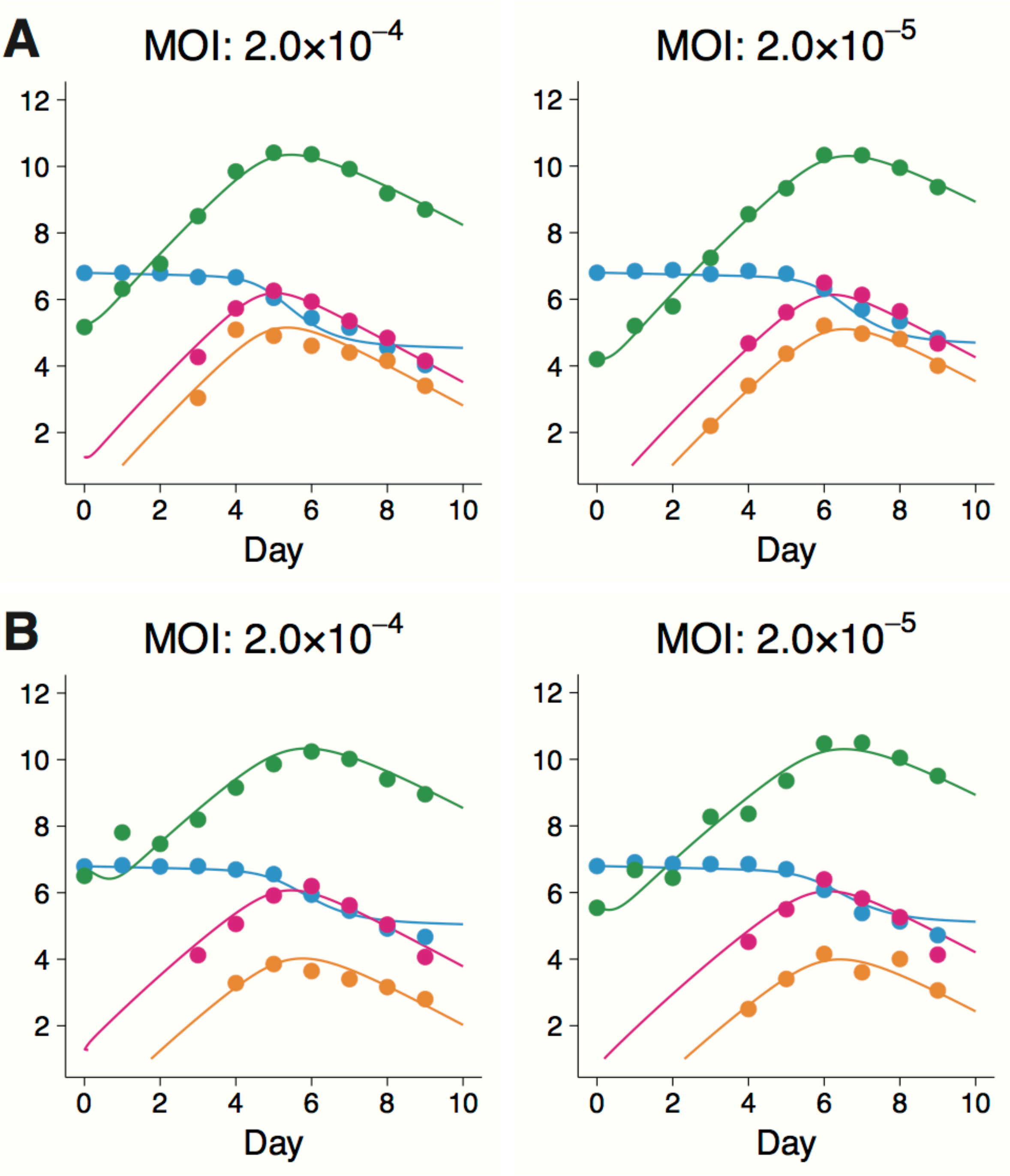

Figure S1 | Dynamics of SHIV-KS661 and -#64 infection in HSC-F cells using nonlinear least-squares regression. We simultaneously fit Eqs. (5-8) to the concentrations of Nef-negative and Nef-positive HSC-F cells and the viral loads consisting of the RNA copies/ml and TCID50/ml for both MOIs of SHIV-KS661 and -#64, respectively, in **A** and **B** using nonlinear least-squares regression that minimizes the sum of squared residuals (SSR). Experimental measurements below the detection limit were excluded when

computing the SSR. The solid curves give the best-fit solution for Eqs.(5-8) and the dots are corresponding to the time-course dataset (log scale): blue, red, green, and yellow represent Nef-negative and Nef-positive HSC-F cells and the viral loads consisting of the RNA copies/ml and TCID50/ml, respectively. The estimated parameters of the model and derived quantities are given in **Table S1**, and the estimated initial values are summarized in **Table S2**.
