# Supplementary material for: A highly pathogenic simian/human immunodeficiency virus effectively produces infectious virions compared with a less pathogenic virus in cell culture
Source: Theor Biol Med Model. 2017 Apr 21;14:9. doi: 10.1186/s12976-017-0055-8 (PMC5401468; doi:10.1186/s12976-017-0055-8)
Supplement: Supplementary file 3 — Parameter values for the in vitro experiment by the nonlinear least squared methods. (PDF 57 kb) [file 12976_2017_55_MOESM3_ESM.pdf]

Table S1. Parameter values for the *in vitro* experiment by the nonlinear least squared methods.

Parameter Name	Symbol	Unit	SHIV-KS661	SHIV-#64
			Value	Value
Parameters obtained from simultaneous fit to full *in vitro* dataset				
Rate constant for infections	β_{50}	$(TCID_{50}/ml \cdot day)^{-1}$	1.45×10^{-5}	1.33×10^{-4}
Death rate of target cells	d	day^{-1}	2.10×10^{-3}	2.10×10^{-3}
Death rate of infected cells	δ	day^{-1}	1.46	1.60
Production rate of total virus	p_{RNA}	RNA copies $\cdot day^{-1}$	3.42×10^{4}	4.34×10^{4}
Production rate of infectious virus	p_{50}	$TCID_{50} \cdot day^{-1}$	0.293	0.0295
