# Supplementary material for: A highly pathogenic simian/human immunodeficiency virus effectively produces infectious virions compared with a less pathogenic virus in cell culture
Source: Theor Biol Med Model. 2017 Apr 21;14:9. doi: 10.1186/s12976-017-0055-8 (PMC5401468; doi:10.1186/s12976-017-0055-8)
Supplement: Supplementary file 4 — Fitted initial (t = 0) values for the in vitro experiment by the nonlinear least squared methods. (PDF 64 kb) [file 12976_2017_55_MOESM4_ESM.pdf]

Table S2. Fitted initial (t=0) values for the *in vitro* experiment by the nonlinear least squared methods.

Variable	Unit	Fitted initial value at MOI of			
		SHIV-KS661		SHIV-#64	
		2×10^{-4}	2×10^{-5}	2×10^{-4}	2×10^{-5}
$T_j(0)$	cells/ml	6.31×10^{6} †	6.31×10^{6} †	6.24×10^{6} †	6.24×10^{6} †
$I_j(0)$	cells/ml	18.5	0.811	18.5	5.04
$V_{RNAj}(0)$	RNA copies/ml	1.52×10^{5}	1.62×10^{4}	5.95×10^{6}	3.91×10^{5}
$V_{50j}(0)$	$TCID_{50}$/ml	0.0307	0.0247	0.154	0.0422

† $T_j(0)$ are fixed.
