# Supplementary material for: A highly pathogenic simian/human immunodeficiency virus effectively produces infectious virions compared with a less pathogenic virus in cell culture
Source: Theor Biol Med Model. 2017 Apr 21;14:9. doi: 10.1186/s12976-017-0055-8 (PMC5401468; doi:10.1186/s12976-017-0055-8)
Supplement: Supplementary file 5 — Distribution of basic reproduction numbers without removal for SHIV-KS661 and -#64 in HSC-F cells. The distributions of the basic reproduction numbers without the effect of removal, $R$R 0=$\beta$β 50 $p$p 50 $T$T(0)/$\delta$δ($c$c RNA+$c$c 50), that were calculated from the es [file 12976_2017_55_MOESM5_ESM.pdf]

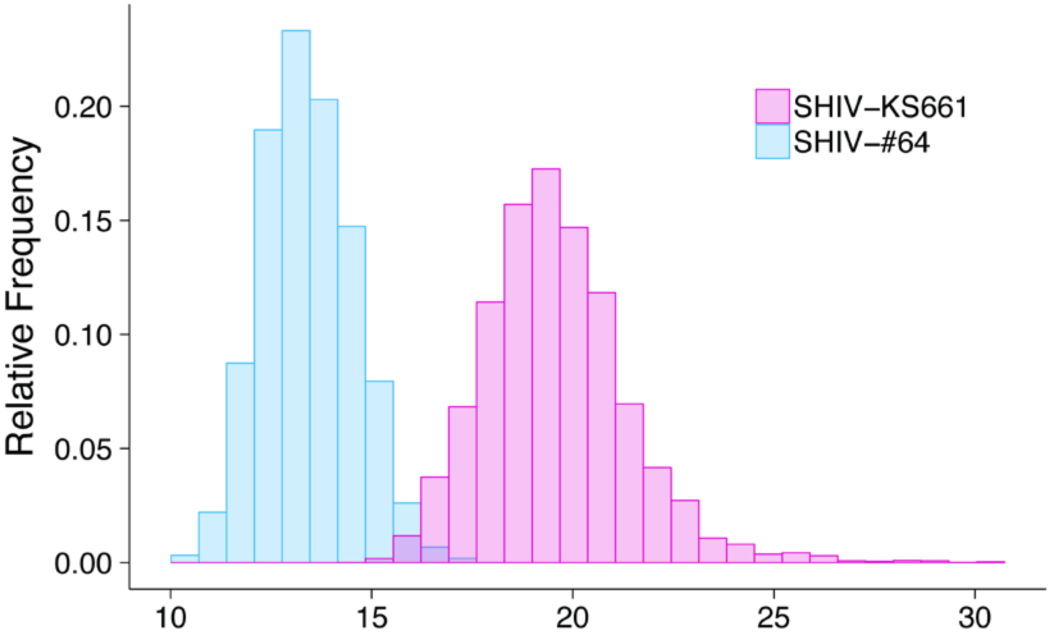

Figure S2 | Distribution of basic reproduction numbers without removal for SHIV-KS661 and -#64 in HSC-F cells. The distributions of the basic reproduction numbers without the effect of removal, $R_0 = \beta_{50} p_{50} T(0)/\delta(c_{RNA} + c_{50})$, that were calculated from the estimated parameter distributions are shown for SHIV-KS661 (red) and -#64 (blue) strains. The basic reproduction number for SHIV-KS661 is significantly different from that for SHIV-#64, as assessed by the repeated bootstrap t-test.
